# Some Tolerance for Fur—Animal Studies in *PLoS Medicine*

**DOI:** 10.1371/journal.pmed.0020203

**Published:** 2005-06-28

**Authors:** The PLoS Medicine Editors

*PLoS Medicine* is a sufficiently new journal that we are often doing something for the first time. This issue's “first” is the publication of a research article that reports data exclusively from animals, more precisely from six cynomolgus macaques used to test the efficacy of a new Lassa fever vaccine. There is no question that animal studies are an important part of medical research, but which ones, if any, belong in a medical journal? More to the point, which ones belong in *PLoS Medicine*? Although our journal's focus is on human studies, we have decided, on occasion, to publish animal studies that have important and proximal implications for clinical research, and maybe even practice.

Lassa fever causes serious morbidity and mortality in West Africa. The virus's natural hosts are rodents, and as there is little chance for effective rodent control in the endemic areas, a vaccine is the most feasible way to gain control of the disease. Several research groups around the world have worked on vaccine development—and their efforts have been boosted by the classification of Lassa virus as a Category A bioweapons agent—but to date no vaccine is available for either general or high-risk application in humans.

Thomas Geisbert and colleagues have developed and now report tests of a recombinant vaccine based on a replication-competent vesicular stomatitis virus (DOI: 10.1371/journal.pmed.0020183). One shot of this vaccine protected four out of four vaccinated monkeys against a lethal virus challenge, whereas the two control animals died, making the vaccine a serious candidate for future application in humans. Clearly many issues about this vaccine still need to be resolved, such as vector safety, duration of protection, and breadth of protection (there are at least four distinct Lassa virus strains). Nevertheless, we accepted this paper because we and our advisers felt that the research was at a stage where clinical questions, such as patient safety and design of early human trials, should inform any additional studies in animals. The proper place for such a study is, we believe, a clinical journal. [Fig pmed-0020203-g001]

**Figure pmed-0020203-g001:**
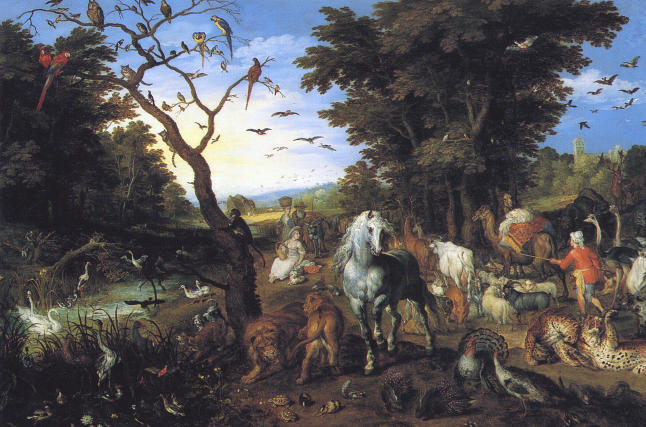
Brueghel's *The Entry of the Animals into Noah's Ark*

There are several other types of animal studies we consider appropriate for publication in *PLoS Medicine*. These include studies that explore off-label uses of approved medical interventions in validated animal disease models, again based on the studies' direct relevance to potential treatment of human patients. More often, we would publish human studies that also include experimental animal data, which typically explore molecular mechanisms suggested by the human data.

Animal studies that are submitted to medical journals can be broadly divided into two groups—“animal clinical trials” and exploratory studies. We will assess the former in a similar way to how we look at human trials. This assessment will include not only the ethical conduct of the study and approval by the respective regulatory authority, but also the rigor of the methodology. Too often, animal clinical trials, i.e., prospective, hypothesis-testing studies that evaluate the effects of a health-related intervention in animals, are not performed with the same rigor that has been developed over past decades and widely adopted by the clinical research community. In particular, animal studies often have inappropriate controls, are underpowered, involve researchers monitoring outcomes who are not blinded to treatment allocation, or lack proper statistical analysis.

Exploratory studies, designed to yield insight into disease etiology, pathology, or the mechanisms by which a particular treatment affects a disease state, are a crucial early part of the translation of basic research findings into clinical practice. However, by and large, they will not be appropriate for *PLoS Medicine*. Instead, we encourage submission of important advances from this early translational stage to *PLoS Biology*, our flagship open-access biology journal (www.plosbiology.org).

It would be foolish to deny the existence of a sizeable “grey area” between these types of study. Between our journals, we will try to provide open-access publication for any important study at this interface between biology and medicine, and will be happy to talk with authors on a case-by-case basis. In addition, we currently cross-reference studies between the two journals—and will do so even more when our new journals come on line. For example, *PLoS Medicine* has published a number of Perspectives on research articles published in *PLoS Biology*, and *PLoS Biology* regularly highlights papers from *PLoS Medicine* on its home page. In this way, because all our journals are open access, the difference to the reader between a paper published in *PLoS Medicine* and any other PLoS journal, is just a rodent click.

